# Clinical Integration of a Smartphone App for Patients With Chronic Pain: Retrospective Analysis of Predictors of Benefits and Patient Engagement Between Clinic Visits

**DOI:** 10.2196/16939

**Published:** 2020-04-16

**Authors:** Edgar L Ross, Robert N Jamison, Lance Nicholls, Barbara M Perry, Kim D Nolen

**Affiliations:** 1 Brigham and Women's Hospital Department of Anesthesiology Pain Management Center Chestnut Hill, MA United States; 2 Brigham and Women's Hospital Harvard Medical School Pain Management Center Chestnut Hill, MA United States; 3 Pfizer, Inc New York City, NY United States

**Keywords:** chronic pain, patient engagement, telemedicine, mHealth, pain measurement

## Abstract

**Background:**

Although many pain-related smartphone apps exist, little attention has been given to understanding how these apps are used over time and what factors contribute to greater compliance and patient engagement.

**Objective:**

This retrospective analysis was designed to help identify factors that predicted the benefits and future use of a smartphone pain app among patients with chronic pain.

**Methods:**

An app designed for both Android and iOS devices was developed by Brigham and Women’s Hospital Pain Management Center (BWH-PMC) for users with chronic pain to assess and monitor pain and communicate with their providers. The pain app offered chronic pain assessment, push notification reminders and communication, personalized goal setting, relaxation sound files, topics of interest with psychological and medical pain management strategies, and line graphs from daily assessments. BWH-PMC recruited 253 patients with chronic pain over time to use the pain app. All subjects completed baseline measures and were asked to record their progress every day using push notification daily assessments. After 3 months, participants completed follow-up questionnaires and answered satisfaction questions. We defined the number of completed daily assessments as a measure of patient engagement with the pain app.

**Results:**

The average age of participants was 51.5 years (SD 13.7, range 18-92), 72.8% (182/253) were female, and 36.8% (78/212) reported the low back as their primary pain site. The number of daily assessments ranged from 1 to 426 (average 62.0, SD 49.9). The app was easy to introduce among patients, and it was well accepted. Those who completed more daily assessments (greater patient engagement) throughout the study were more likely to report higher pain intensity, more activity interference, and greater disability and were generally overweight compared with others. Patients with higher engagement with the app rated the app as offering greater benefit in coping with their pain and expressed more willingness to use the app in the future (*P*<.05) compared with patients showing lower engagement. Patients completing a small number of daily assessments reported less pain intensity, less daily activity interference, and less pain-related disability on average and were less likely to use the two-way messaging than those who were more engaged with the pain app (*P*<.05).

**Conclusions:**

Patients with chronic pain who appeared to manage their pain better were less likely to report benefits of a smartphone pain app designed for chronic pain management. They demonstrated lower patient engagement in reporting their daily progress, in part, owing to the perceived burden of regularly using an app without a perceived benefit. An intrinsically different pain app designed and targeted for individuals based on early identification of user characteristics and adapted for each individual would likely improve compliance and app-related patient engagement.

## Introduction

### Background

Pain is a major reason that individuals seek health care treatment, and it is estimated that more than 25 million US adults are affected by daily pain [1]. Chronic pain is known to impose a tremendous burden on the quality of life of the affected individuals [2]. According to the Global Burden of Disease Study of the Institute for Health Metrics and Evaluation and the World Health Organization, chronic pain has consistently been ranked first in associated disability and overall burden between 1990 and 2017 [3]. It has been determined that chronic pain adversely affects individuals at a higher frequency than depression, substance abuse, and Alzheimer disease [4,5]. An influential report by the Institute of Medicine on *Relieving Pain in America* highlighted the urgent need for the development of better methods for tracking and treating pain because of the ever-increasing costs associated with this condition [6].

Innovative technology can be used by health care providers to track persons with chronic pain, engage the patients between clinic visits, and offer information and support to improve coping. There has been a rapid increase in smartphone apps used to monitor and record health data partly due to the increase in mobile device availability [7]. According to the Pew Research Center, about three-quarters of US adults (77%) stated that they owned a smartphone, and 46% of these owners said that their smartphone is something “they could not live without” [8,9]. Individuals living in both urban and rural communities are capable more than ever of monitoring their progress and sending information directly to their health care providers using sophisticated apps [10].

There is evidence that tracking real-time data using momentary ecological assessment is preferable to retrospective diary entry [11-14]. Apps using innovative time-stamped technology can be particularly helpful in tracking variations in pain intensity and other health-related symptoms between clinic visits [7,15,16]. Large datasets of daily pain assessment offer opportunities for the employment of computer-based classification and artificial intelligence [17]. Various available smartphone apps target people with both non–cancer- and cancer-related pain [18-21]. Although many of these apps are commercially accessible, most of them (approximately 86%) have been found to lack professional medical involvement in their development [22]. Lallo et al [23] reviewed 224 pain apps and found little evidence that health care professionals had been involved in creating the apps. The authors also found that only 2% of the apps they reviewed incorporated interactive social support and goal setting. None of the apps that were reviewed contained the recommended five main categories of functionality: the ability to self-monitor, set goals, build skills, educate, and provide social support.

In a more recent review, Bhatterai et al [22] examined 373 pain self-management apps; only 4 successfully met their inclusion criteria according to an established usability evaluation tool. In another recent review of 195 pain management apps, Portelli and Eldred [24] found only 6 apps that incorporated a specific psychological component. The authors concluded that existing pain apps were often constructed by software developers with little input from health care professionals and patients with pain. They also reported that the pain apps tend to contain minimal theoretical content for facilitating self-management or behavioral change. Unfortunately, the life expectancy of most smartphone apps is brief. Three-fourths (75%) of users discontinue using an app within 48 hours of downloading it, and 25% of apps are discarded after the first opening [25]. On the basis of anonymized data points from more than 125 million mobile phones, it is estimated that 80% of apps fade away in time frames as short as 72 hours, and 21% of users use an app only once [26,27].

### Objective

The purpose of this analysis was to determine the long-term effects of using a smartphone pain app that offers pain management strategies and allows patients with chronic pain to assess, monitor, and communicate their condition to their health care providers*.* We were particularly interested in learning from quantitative and qualitative feedback from users about factors that might contribute to improved patient engagement and what might affect adherence to using a smartphone pain app between clinic visits. We were interested in identifying the type of user who would commit to continuing to use a pain app in the future. Finally, we examined qualitative feedback from the users to help identify ways to improve a smartphone pain app.

## Methods

### Design

**Figure 1:**
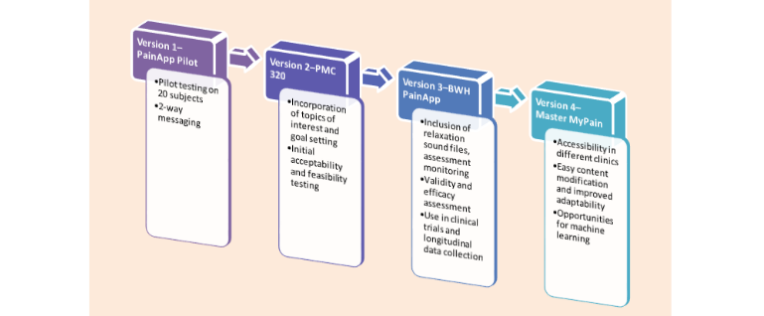
Key development highlights from each version of the pain app. BWH: Brigham and Women's Hospital; PMC: Pain Management Center.

**Figure 2:**
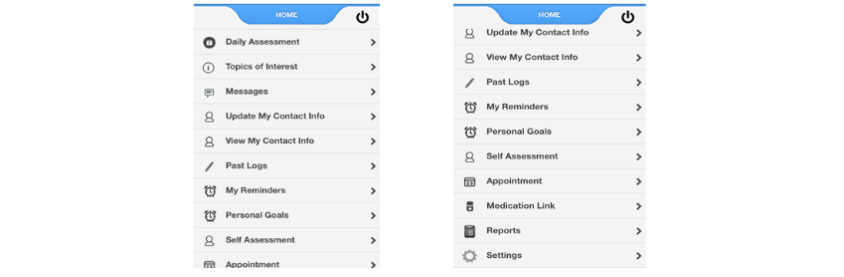
Pain app version 3 home page with links when scrolled down.

This is a retrospective analysis of data gathered from a smartphone pain app designed by Brigham and Women’s Hospital Pain Management Center (BWH-PMC) to assess longitudinal combined information about satisfaction and compliance with the use of a smartphone pain app for persons with chronic pain over 3 months. The analysis plan was approved by the hospital’s internal review board. A team from the BWH-PMC helped develop and test multiple versions of a smartphone pain app used on iOS and Android devices. Initial input from 20 patients with chronic pain was obtained to assist in the development process of the first version of the app (PainApp Pilot; Figure 1). The pain app was tested for security, and all data were saved on a secure encrypted password-protected server. All versions of the pain app were tested and uploaded to the Apple iTunes Store and Google Play Store. A validated version of the app used for this analysis (BWH PainApp) could be downloaded for free and could be used to monitor progress and provide feedback to the user through two-way messaging (Figure 2).

**Figure 3:**
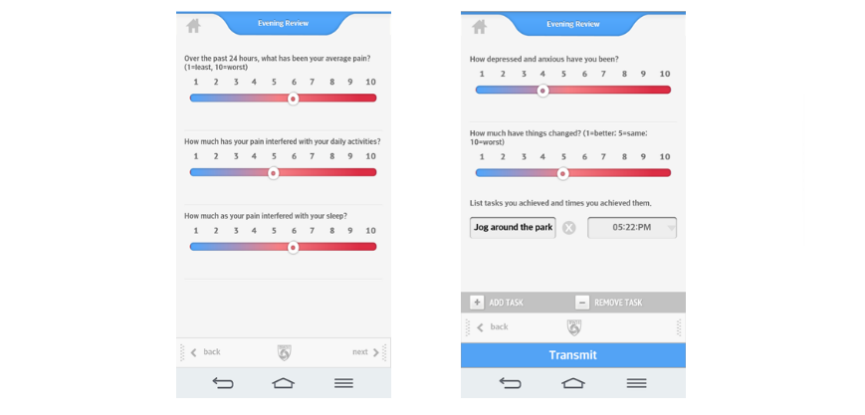
Pain app version 3 daily assessments and goal-setting tasks.

Data on the server were available only to BWH-PMC personnel through a secure password-protected administration portal. Components of the smartphone app included demographic and contact information, a comprehensive chronic pain assessment, 5-item daily assessments with push notification reminders (Figure 3), personalized goal setting, relaxation sound files, topics of interest with psychological and medical pain management strategies, and line graphs from the daily assessments that could be saved and placed on the patients’ electronic medical record (EMR).

Data were collected by BWH-PMC from a series of studies using the third version of the smartphone pain app (BWH PainApp) between February 2015 and May 2018 among patients with noncancer-related chronic pain. Previously conducted study methods have been reported earlier by BWH-PMC [28-31]. Subjects were recruited either by invitation to participate in a randomized trial by their treating physician, or they responded to a flyer left in clinic waiting rooms. The trials were designed to investigate the efficacy of a pain app [28,29] and the efficacy of devices to help manage pain (eg, vibrating gloves and a transcutaneous electric nerve stimulation device) [30,31]. Interested and eligible participants signed a consent form and completed baseline measures through these previously conducted studies. Subjects were asked to complete a packet of questionnaires at baseline and again after a 3-month follow-up. The pre-post questionnaires were completed on paper, and data were transferred to an electronic database. Data were also captured from baseline and daily assessments from the pain app. All patients were asked to inform the investigators if any unforeseen medical changes occurred. Subjects were informed about how to find the app (either on the iTunes Store or Google Play Store) and were assisted in downloading the pain app if assistance was needed. Support was offered to address any technical problems that the subject might have encountered. Most of the on-boarding process was done live with a research assistant (RA), and additional assistance was offered with two-way messaging on the administrator portal. Subjects were also able to contact the RA if and when they encountered any technical problems.

All participants were encouraged to complete a 5-item daily assessment on the pain app about their pain, sleep, mood, activity interference, and whether they had gotten better or worse on a visual analog scale (Figure 3). Participants were instructed to complete the assessments around the same time each day for 3 months (although some participants continued to use the app beyond 3 months). Line graphs of the data were made available on the server for subjects. These reports could be copied and pasted onto the subject’s hospital EMR. The app would sometimes have a lag in transmitting data to the server, and as such, subjects did not always get to see their summarized data on these line graphs. Participants involved in a specific study received US $25 after completing the baseline packet of questionnaires, and US $50 after completing the 3-month assessments. Compensation was based on the completion of the follow-up questionnaires regardless of app usage.

### Participants

BWH-PMC recruited patients with chronic pain to participate in 1 of 4 published studies [28-31]. Participants needed to be 18 years or older and own a compatible smartphone (iOS or Android device). Other inclusion criteria included (1) having chronic pain for more than 6 months, (2) averaging 4 or greater on a pain intensity scale of 0 to 10, and (3) able to speak and understand English. Patients were excluded if they had (1) any cognitive impairment that would prevent them from understanding the consent, study measures, or procedures, (2) any clinically unstable medical condition judged to interfere with study participation, (3) a pain condition requiring urgent surgery, (4) a present psychiatric condition (eg, Diagnostic and Statistical Manual of Mental Disorders diagnosis of schizophrenia, delusional disorder, psychotic disorder or dissociative disorder) that was judged to interfere with the study, (5) visual or motor impairment that would interfere with the use of a smartphone, and (6) an active addiction disorder over the past 6 months (positive on the Mini International Neuropsychiatric Interview, v.5.0) [32] that would interfere with study participation.

### Measures

Acceptability, tolerability, feasibility, and effectiveness of the third version of the pain app were assessed by examining the number and frequency of daily assessments, the number of subjects who continued to use the app after the initial download, and the numeric and qualitative satisfaction ratings. Any reported safety issues were also documented. Overall outcome efficacy was determined through standardized paper-based measures administered at baseline and again after 3 months from this baseline assessment [28-31].

Pain intensity and pain description were assessed using the Brief Pain Inventory (BPI) [33]. This self-report questionnaire, formerly the Wisconsin Brief Pain Questionnaire [34], has shown sufficient reliability and validity. Scale (rated from 0=no pain to 10=worst pain possible) indicates the intensity of pain at its worst, least, average, and pain now. A figure representing the body was used for the patient to shade the area corresponding to his or her pain. Test-retest reliability for the BPI ratings of pain revealed high correlations of 0.93 for worst pain, 0.78 for usual pain, and 0.59 for pain now [33].

Activity interference and disability was assessed with items from the BPI and the Pain Disability Inventory (PDI) [35]. The PDI is a 7-item questionnaire rated from 0 to 10 on the level of disability of 7 areas of activity interference including family or home responsibilities, recreation, social activity, occupation, sexual behavior, self-care, and life-supporting behaviors. It has shown to have excellent test-retest reliability and validity and is sensitive to high levels of disability [35].

Mood, negative affect, and emotional distress were assessed using the Hospital Anxiety and Depression Scale (HADS) [36,37] and certain initial baseline questions on the pain app. The HADS is a 14-item scale that helps determine the presence and severity of anxiety and depression. Each item is coded from 0 to 3 (eg, not at all, most of the time) with 7 items assessing anxiety and 7 items measuring depression. The HADS has adequate reliability (Cronbach alpha=.83) and validity [37]. We also examined the construct of catastrophizing using the Pain Catastrophizing Scale (PCS) [38,39]. The PCS is a 13-item self-report measure that examines pain rumination, magnification of symptoms, and general helplessness. The responses range from not at all (0) to all the time (4) on a 5-point scale. The PCS is found to predict levels of pain and distress among clinical patients and has good psychometric properties with excellent reliability (Cronbach alpha=.87-.95) and validity [17,40]. Total scores of 30 or greater represent a clinically relevant level of catastrophizing (75th percentile).

After 3 months, participants were asked to respond to a 5-item paper-based satisfaction questionnaire designed to investigate the perceived benefit of how easy the pain app was to use and navigate, how useful the daily reminders were, how much the program helped them cope with their pain, and how willing they would be to use the pain app in the future. All items, which were developed in a previous study [29] and adapted from a previously validated measure [41], were rated on a 0 to 10 scale (0=worse/not at all helpful to 10=best/very helpful).

### Statistical Analysis

This retrospective analysis was conducted by BWH-PMC. Univariate and multivariate descriptive analyses were performed on all the dependent variables at baseline and at follow-up. Chi-square, *t* tests, interitem correlations, exploratory factor analyses, and canonical discriminant function analyses were conducted as appropriate using SPSS (version 25.0, IBM Corporation) [42,43]. Subjective comments about the use of the pain app were also collected and summarized.

## Results

### Patient Demographic Characteristics

A total of 253 patients with chronic pain were engaged by BWH-PMC to use a revised third version of the smartphone pain app. The average age of patients was 51.4 years (SD 13.7, range 18-92); 73.1% (185/253) of patients were female and 82.9% (209/252) of patients were white (Table 1). Pain duration averaged 11.8 years (SD 10.7), and 36.8% (78/212) of patients reported having primary low back pain. Most of the patients (n=243) were taking prescription medication at the time of the study, and 39.9% (97/243) were prescribed opioids for pain. Most subjects were overweight with a BMI averaging 30.1 kg/m^2^ (SD 7.4). Most of the subjects (171/253, 67.6%) had iPhones, whereas 32.4% (82/253) of the subjects had Android smartphones.

**Table 1:** Patient demographic characteristics (N=253).

Variable		Value	Range
Age (years), mean (SD)		51.5 (13.8)	18-92
Gender, female, n (%)		182 (72.7)	N/A^a^
**Ethnicity, n (%)**			
	White	206 (82.7)	N/A
	African American	16 (6.4)	N/A
	Hispanic	17 (6.8)	N/A
	Other	10 (4.0)	N/A
Pain duration (years), mean (SD)		11.8 (10.7)	0.5-50
**Pain site, n (%)^b^**			
	Low back	78 (36.8)	N/A
	Multiple sites	77 (36.3)	N/A
	Cervical/upper extremity	31 (14.6)	N/A
	Lower extremity	11 (5.2)	N/A
	Abdominal/pelvic	13 (6.1)	N/A
	Head/face	2 (0.9)	N/A
**Pain intensity^c^, mean (SD)**			
	Worst pain	7.7 (2.1)	1-10
	Least pain	3.3 (2.3)	0-10
	Average pain	5.4 (1.8)	1-10
Depth of pain, mean (SD)		203.3 (48.0)	0-270
Take opioid medication, n (%)^d^		97 (39.9)	N/A
BMI (kg/m^2^), mean (SD)		30.1 (7.4)	12.2-54.7
Number of times wake during night, mean (SD)		2.6 (2.1)	0-10
Sleep hours, mean (SD)		6.3 (1.8)	1-12
Pain interference (total)^e^, mean (SD)		4.9 (2.7)	0-10
Pain Disability Index, mean (SD)		31.5 (17.7)	0-70
Hospital Anxiety and Depression Scale total, mean (SD)		14.9 (7.7)	0-36
Pain Catastrophizing Scale, mean (SD)		17.3 (12.2))	0-50
Number of symptoms^f^ (present or absent), mean (SD)		1.6 (2.4)	0-13
Number of pain descriptors^g^ (present or absent), mean (SD)		4.1 (1.9)	1-9
Number of daily assessments, mean (SD)		62.0 (49.9)	1-426

^a^N/A: not applicable.

^b^N=212.

^c^0=no pain; 10=pain as bad as you can imagine.

^d^N=243.

^e^During the past 24 hours, how much has your pain interfered with (1) general activity, (2) mood, (3) walking ability, (4) normal work, (5) relations with others, (6) sleep, and (7) enjoyment of life? 0=has not interfered; 10=completely interfered.

^f^Side effect symptoms: constipation, dizziness, dry mouth, headache, itching, memory lapse, confusion, nausea, nightmares, sneezing, sweating, visual problems, weakness, and other.

^g^Pain descriptors: throbbing, stabbing, aching, burning, pricking, pulling, shooting, numbing, and other.

### Patient Engagement Results

Of the 253 subjects considered for the analysis, 43 (18.1%) reported some type of technical problem with the app during the study period that briefly restricted their daily assessments. This did not significantly affect their engagement with the pain app. The total number of daily assessments from the pain app averaged 62.0 (SD 49.9). Comparisons between baseline measures and repeat measures at 3 months showed an overall decrease in average pain intensity on the BPI (5.3, SD 1.8 vs 4.9, SD 2.3; t_185_=4.0; *P*<.001) and a decrease in disability on the PDI (30.6, SD 17.7 vs 27.2, SD 18.2; t_156_=3.9; *P*<.001), but no differences in mood (mean HADS score 14.4, SD 8.1 vs 14.6, SD 8.3) and pain catastrophizing (mean PCS score 15.7, SD 1.3 vs 16.3, SD 12.8).

A total of 72.3% (183/253) users completed the satisfaction questionnaire after approximately 3 months. No significant differences in demographic characteristics were found between those who completed the satisfaction questionnaire and those who did not complete this questionnaire. Most users found the app easy to use (mean 8.7, SD 2.2) and easy to navigate (mean 8.5, SD 2.4; 0=not at all easy; 10=very easy). The majority of users also found the daily reminders to be useful (mean 6.7, SD 3.9; 0=not at all useful, 10=very useful). Some of the users, primarily the Android users, reported that the push notification reminders did not consistently work on their phone, and they were more likely to rate lower perceived usefulness of the daily reminders because they did not work. The users felt that the app offered some help in coping with their pain (mean 4.5, SD 3.7; 0=not at all helpful, 10=very helpful), whereas the majority of the users felt that they would be willing to use the app in the future (mean 7.1, SD 3.3; 0=not at all willing, 10=very willing).

No significant differences were found on demographic variables of age, gender, ethnicity, or pain duration on all outcome variables. Those who reported liking the pain app were more likely to use it often to submit more daily reports and reported greater pain intensity and more disability. Pearson product-moment correlations between the 5 satisfaction questions ranged between 0.21 and 0.58.

Factor analysis of the satisfaction questionnaire responses using principal component analysis with Varimax rotation found two factors above an eigenvalue of greater than 1.0: (1) easy to use, easy to navigate, useful reminders (correlation *r*=0.48; eigenvalue=2.47) and (2) helped to cope with pain and would use the app in the future (*r*=0.51; eigenvalue=1.01). The first factor, containing 3 of the satisfaction questions, was labeled *easy to use*. The second factor, containing the other two satisfaction questions, was labeled *help with coping and future use.* Those who felt that the app helped them in coping also were more likely to report that they would use the pain app in the future. Combined, these 2 factors accounted for 70.0% of the variance.

Pearson product-moment correlations were run between the combined satisfaction ratings of *easy to use* (satisfaction questions 1, 2 and 3) and *help with coping and future use* (questions 4 and 5) and the number of daily assessments, BPI activity interference, and PDI scores at 3 months (Table 2).

Discriminant function analyses were run using those variables, which revealed significant differences between those with generally higher ratings on *help with coping and future use,* compared with those with lower ratings on these items. Three items were identified using stepwise entry: (1) total number of messages sent and received, (2) total PDI baseline scores, and (3) BMI score (Wilks Lambda=0.89; *P*<.001) and correctly classifying 69.4% of the cases entered. This means that, overall, these 3 variables would correctly classify someone approximately 70% of the time as to the app being helpful to cope with pain and used in the future.

Differences were examined on the baseline and outcome variables between those selected patients with pain who felt that the pain app both helped them cope with their pain and were willing to use the app in the future (n=84; >7/10) and those who reported that the pain app both did not help them cope and were less inclined to use it in the future (n=81; <7/10) based on the 3-month satisfaction questions (Table 3). No differences were found between groups in age, gender, pain site, ethnicity, or pain duration. Those who rated the pain app more favorably reported higher pain ratings (baseline and follow-up), more activity interference, more pain-related disability, used more words to describe their pain, reported more side effects, had higher BMI scores, and were more often taking opioids for their pain than those who were less favorable about the pain app (*P*<.05). Those who felt that the app helped them cope with their pain and would use the app in the future completed more daily assessments and used the two-way messaging service on the app more than those who felt that the app was not as helpful (*P*<.05).

Most of those who responded to the follow-up question *Is there anything about the pain app that you would change?* had no comments (eg, no, none, not really, not sure), and there were a number of positive comments (eg, I found it easy to use, Thanks for the opportunity to use the app). Examples of negative feedback and specific comments about difficulties encountered with the push notification and recommendations for improvements are included in Multimedia Appendix 1. Some users encountered a number of difficulties with the functionality of the app (slow, not accurate, problems with deleting password) and expressed difficulties in seeing past logs and concerns about being constantly reminded about their pain. Some pointed out the challenges they experienced when updating their phones or changing their phone carriers. Many expressed problems they encountered with daily push notification reminders. This was found to be particularly prevalent among Android device owners. Other requests included making the pain app more adaptive to each user’s specific condition, adding more instruction when starting to use the app, giving a clear indication when a daily assessment was completed, being able to continue to listen to the relaxation sound files even when the app is closed, and incorporating clearly designated areas to type in free text that can be sent to providers.

**Table 2:** Pearson product-moment correlations among patient satisfaction questionnaire responses between those who found the pain app easy to use, and those who felt that the app helped them to cope and would be willing to use the pain app in the future (0=very satisfied; 10=very unsatisfied).

Variable		Pearson product-moment correlations for *easy to use*	Pearson product-moment correlations for *helped cope and future use*
Age (years)		0.20^a^	−0.08
**Brief Pain Inventory (0-10)**			
	Worse pain	−0.06	0.18^a^
	Least pain	0.15	0.22^b^
	Average pain	0.10	0.24^b^
BMI (kg/m^2^)		0.11	0.26^b^
Brief Pain Inventory activity interference (0-10)		−0.03	0.24^b^
Pain Disability Inventory total (0-70)		0.00	0.19^a^
Side effect list total (0-14)^a,c^		−0.18^a^	0.19^a^
Pain description total (0-9)^b,d^		−0.23^a^	0.15
Number of daily assessments entered		0.09	0.15
Total number of messages sent and received		−0.01	0.18^a^
Opioids (yes/no)		0.11	0.19^a^

^a^*P*<.05.

^b^*P*<.01.

^c^Side effect symptoms: constipation, dizziness, dry mouth, headache, itching, memory lapse, confusion, nausea, nightmares, sneezing, sweating, visual problems, weakness, and other.

^d^Pain descriptors: throbbing, stabbing, aching, burning, pricking, pulling, shooting, numbing, and other.

**Table 3:** Differences between patients with pain who felt that the pain app helped them cope with their pain and were willing to use the app in the future (n=84) and those who reported that the pain app did not help them cope and were less inclined to use it in the future (n=81).

Variable^a^	Yes (n=84)	No (n=81)	*t* test (*df*)	Chi-square (*df*)
BPI^b^ pain (baseline, range 0-10), mean (SD)	5.7 (1.9)	5.1 (1.8)	2.3 (157)^c^	N/A^d^
BPI pain (3-month follow-up, range 0-10), mean (SD)	5.2 (2.4)	4.4 (2.0)	2.3 (155)^c^	N/A
BPI activity interference (range 0-10), mean (SD)	5.0 (2.4)	3.9 (2.6)	2.7 (157)^c^	N/A
BPI activity interference (3-month follow-up, range 0-10), mean (SD)	4.7 (2.8)	3.8 (2.8)	Not significant	N/A
PDI^e^ total (baseline), mean (SD)	34.6 (16.9)	27.2 (17.0)	2.7 (145)^f^	N/A
PDI total (3-month follow-up), mean (SD)	31.8 (18.3)	25.0 (17.0)	2.4 (150)^c^	N/A
Pain description (range 0-9)^g^, mean (SD)	4.5 (2.1)	3.9 (1.6)	2.1 (160)^c^	N/A
Side effects total (yes, range 0-14)^h^, mean (SD)	7.8 (15.6)	4.1 (8.6)	Not significant	N/A
BMI (kg/m^2^), mean (SD)	31.6 (7.8)	28.1 (6.5)	3.2 (160)^f^	N/A
Opioids (% yes of total)	22.6	16.4	N/A	4.3 (1)^c^
Number of daily assessments entered, mean (SD)	83.6 (62.3)	65.9 (37.9)	2.2 (162)^c^	N/A
Total messages, mean (SD)	13.1 (12.3)	8.9 (7.9)	2.6 (162)^c^	N/A

^a^No differences were found between groups on age, gender, pain site, ethnicity, or pain duration.

^b^BPI: Brief Pain Inventory.

^c^*P*<.05.

^d^N/A: not applicable.

^e^PDI: Pain Disability Inventory.

^f^*P*<.01.

^g^Pain descriptors: throbbing, stabbing, aching, burning, pricking, pulling, shooting, numbing, and other.

^h^Side effect symptoms: constipation, dizziness, dry mouth, headache, itching, memory lapse, confusion, nausea, nightmares, sneezing, sweating, visual problems, weakness, and other.

## Discussion

### Principal Findings

Although many pain-related apps exist, attention has been given recently to understanding how these apps are used over time and what factors contribute to greater compliance and patient engagement [44,45]. This study examined factors that contributed to increased patient engagement in using a smartphone pain app. Overall, the pain app was found to be usable and easily accepted among most of the users and, based on the 3-month follow-up assessments, most of the users reported improvement in pain intensity and activity interference. However, those rating the app as easy to use did not necessarily report that the app improved their ability to cope with the pain or that they would necessarily continue to use the app in the future. Demographic variables such as age, gender, or ethnicity were not found to play a role in predicting overall improvement, compliance or satisfaction with use of the pain app. Throughout the study, those persons with chronic pain who reported higher pain intensity and greater pain-related disability were found to like the app more, use the app more, and express greater willingness to use the app in the future compared with those who were less disabled because of their pain. This suggests that the ability to tailor the app to meet the needs of each user could have an important effect on improving compliance. These analyses also suggest that apps should be selectively assigned to those who may present with certain indicators signaling a greater likelihood of benefiting from a pain app to cope with their pain.

The challenge with mobile health (mHealth) technology is to encourage and motivate participants to continue to use an app to track behavior, maintain contact with their provider, and make improvements in their condition. This is particularly important among individuals with chronic illnesses. The goal of innovative mHealth technology is to offer medical and psychological assistance remotely to reduce health care utilization by reducing clinic and emergency room visits and unnecessary expensive tests. This is a future direction for health care technology, but engaging individuals in ways that increase use of this technology continues to represent a challenge among app developers. It may be no surprise that those patients with pain who used the app more were more satisfied with the pain-related software program. It is interesting to speculate why those with more pain, greater self-reported disability, greater weight, more use of opioids, and more pain descriptors were more satisfied with the smartphone pain app. Quite possibly, those who were busy throughout the day found the app to be more bothersome. Subjective feedback suggests that some preferred not to focus on their pain and found the frequent monitoring to be more of a bother than helpful. Those who reported more limitations owing to their pain might have been more focused on their pain and welcomed the opportunity to share their experience with their providers. Some may have also wanted to verify their disability and document their limitations for others.

There are many challenges with pain apps going forward. Few physicians recommend pain apps because of lack of time, lack of information about which apps are reliable, concerns of liability, and insufficient evidence that the use of an app will improve outcomes [46]. Even if a physician recommends an app to a patient, there is no guarantee that the patient will download it, use it, and continue to use it. Patients need to have the desire to self-manage, and the role of patient engagement is vitally important. Monitoring data with stand-alone apps that collect data but make it difficult to share with providers will reduce the chance that the apps will be used. In addition, physicians do not have time to wade through raw data, so analytics are needed to help make the data digestible. Providers will also not spend time to open a website to view data; thus, there needs to be an easy way to incorporate pain app data into an EMR system.

There are a number of limitations of this analysis that should be highlighted. As with any new technology, we encountered some software and hardware difficulties that may have adversely affected the use of the app and consequently affected the outcome data. Some subjects did not receive reminders or push notifications to complete their daily assessments, which seemed to be reported mostly by Android smartphone owners. In addition, some encountered difficulties when they upgraded their smartphones, including problems downloading the program to their new device. They also reported minor problems with the app when software updates were made to either the iPhone or Android devices. Corresponding changes were needed in the software code of the pain app every time these changes were made to the iOS and Android platforms. The BWH-PMC staff also needed to make periodic changes to the administrative portal and server, which caused delays in capturing patient data. Thus, factors other than patient noncompliance, including technical difficulties with the software and the devices, may have accounted for the perceived benefit from the pain app. Not all users were able to participate owing to the limitations of their phone capabilities or them not owning a smartphone. Thus, these results may have been affected by selection bias. Patients were encouraged to use the app as part of a study, which may have influenced the use of the app more than what might have been done if patients were not involved in a study. We also could not determine how the availability of RA support was an influencing factor in engagement. It should also be pointed out that the results are correlational in nature, and no causal relationships can be assumed.

### Conclusions

This retrospective analysis demonstrates that a smartphone pain app for persons with chronic pain can be perceived to be easy to use, but certain factors, including greater pain and disability, might have an increased influence in motivating individuals to use the app. It also highlights potential challenges in using mHealth technology. Future improvements are needed to make pain apps more adaptive and engaging and directly tailored to the individual user. This would likely have a positive impact on adherence and may lead to increased improvements among persons with chronic pain.

## Appendix

Multimedia Appendix 1 Comments regarding use of the pain app after a 3 month trial "Is there anything about the pain app that you would change.".

## Abbreviations

AMIA: American Medical Informatics Association
BPI: Brief Pain Inventory
BWH-PMC: Brigham and Women’s Hospital Pain Management Center
EMR: electronic medical record
HADS: Hospital Anxiety and Depression Scale
mHealth: mobile health
PCS: Pain Catastrophizing Scale
PDI: Pain Disability Inventory
RA: research assistant
