# Supplementary material for: Clinical Integration of a Smartphone App for Patients With Chronic Pain: Retrospective Analysis of Predictors of Benefits and Patient Engagement Between Clinic Visits
Source: J Med Internet Res. 2020 Apr 16;22(4):e16939. doi: 10.2196/16939 (PMC7193441; doi:10.2196/16939)
Supplement: Multimedia Appendix 1 [file jmir_v22i4e16939_app1.docx]

**Table 4.** Comments regarding use of the pain app after a 3-month trial “Is there anything about the pain app that you would change?”^a^

| **Number** | **Below are examples of generally negative comments.** |
| --- | --- |
| 1 | Not sure if I would use the pain app in the future. I disliked the constant reminder about my pain. I believe this does not help. When you click to put in your password, the app can move up so you can accidentally hit login and then it tells you that your password is incorrect. |
| 2 | The scale was sometimes hard to move at the end of the ranges. For example, it was hard to get it to 0-1 or 9-10. Also, I would have it not send a reminder if the survey was already filled out for the day. |
| 3 | I personally had issues trying to view old assessments but that's it! I mostly wanted to see them when I wasn't sure if I had done my assessment that day. It just didn't show me what I entered in the search box. |
| 4 | I don’t think this app is ready for prime time. |
| 5 | I wouldn't know why anyone would use it. I found it was a little slow uploading the pages. |
| 6 | It is too easy to delete the password. |
| 7 | You need to improve the ability to record when tasks are competed. It frequently didn't work for me. |
| 8 | I wish all the features of the pain app were working. It's design is very promising. |
| 9 | Some aspects of it did not work well. I could not accurately look up past logs and the dates were consistently wrong. Sometimes the message received when transmitting appeared confusing. Perhaps a little more practice on it initially might have been helpful before actually having to use it. |
| 10 | This app needs updates. Often, but not always, my app would not reset the numbers back to zero and would remain from the day before. |
| 11 | It isn't too intuitive. I'm used to using a Mac. |
| 12 | If I forgot to enter my daily assessment, I could not go and back date. |
| 13 | When you press the back button or come off the app, it closes all the way and it has to re-log back on and you lose your space. I wonder if it can just stay on the page (or question) where you left off. |
| 14 | I changed my phone from iPhone to Samsung and I had to re-download the pain app. |
| 15 | I got a new phone and I downloaded the pain app again and had to redo all of the initial evaluation. |
| 16 | It would only let me put in 2 activities in a day, so I stopped putting them in. If there were more, I could better track whether I went for a walk or drove or shopped, etc. each day to better judge how much the pain directly affects my life. |
|  |  |
|  | **Below are examples of comments regarding problems with the push notification.** |
| 17 | The daily reminder alarm never worked on my phone. |
| 18 | Daily reminder feature did not work. Would also add a feature that allows you to see that you have already completed your self-assessment for the day. Really would have liked to see the "pain graph" feature working. |
| 19 | The daily reminder feature didn't work. Incorporate use of the phone's back button so the user can avoid having to use the home icon in the app. |
| 20 | Make it more friendly for Android users. Daily reminder didn't work. |
| 21 | The notification did not always go off. |
| 22 | We set up the reminder for mid-day, but it went off in the afternoon. Get the reminder to go off at the right time. |
| 23 | The pain app is not notifying me about the daily assessments. |
| 24 | The daily reminders didn't work. A way to see the dates you entered into the app would be helpful to check submissions and would help if you don't remember if you already completed your entry that day. |
|  |  |
|  | **Below are examples of recommendations for Improvements.** |
| 25 | In general, make it easier to use. |
| 26 | You have to answer the same questions each day. Consider changing the questions. |
| 27 | The reminders need to be more obvious. |
| 28 | There needs to be a way to set up reminders for more than one day. |
| 29 | I think you should add more quantitative and qualitative questions relating to the specific areas of where the pain is. I did not find the #4 and #5 questions as useful, at least for me. I feel like it could have been more descriptive with different questions. I liked there were 5 daily questions, but I would have been fine with up to 8! |
| 30 | Another reminder would be helpful if I had forgotten to input the daily pain assessment. |
| 31 | The music should last longer. |
| 32 | The question "How did your pain interfere with", I would change to "Did you feel pain when you…" While I never let my pain "interfere," i.e. stop my activity, I did feel pain during these times. |
| 33 | The questions asked were somewhat general and not relevant, maybe restructure the questions. |
| 34 | Add an introduction that explains each category. Are there privacy settings available to the patient? |
| 35 | Sometimes it doesn't save my daily assessment responses. I had to check it at night to make sure the responses are still there. Otherwise, it's great! |
| 36 | Ensure it's working 24/7. Make audio files stay on when you turn off your screen. Ensure you can enter more than 2 activities per day. Have the home page let you know when you have messages. If you read a message, ensure the "inbox" label no longer reflects that you have a message. |
| 37 | It should let you see your previous ratings so you know if anything changed. Also, include better reminders and have the graphs available on the app. |
| 38 | More visibility and information whether you already filled out the daily questions. |
| 39 | There were times when the app stopped working. Also, there should be a section to enter free text. |
| 40 | Have a recognizable voice on the app from someone in the clinic to help me with the breathing techniques. |

^a^There were many written comments of “no,” “not really,” “nothing,” “none,” and “not sure.”
